# Correlation transmission of spiking neurons is boosted by synchronous input

**DOI:** 10.1186/1471-2202-12-S1-P144

**Published:** 2011-07-18

**Authors:** Matthias Schultze-Kraft, Markus Diesmann, Sonja Grün, Moritz Helias

**Affiliations:** 1 Machine Learning Group, Berlin Institute of Technology, Berlin, Germany; 2 Laboratory for Computational Neurophysics, RIKEN Brain Science Institute, Wako City, Japan; 3 Brain and Neural Systems Team, RIKEN Computational Science Research Program, Wako City, Japan; 4 Institute of Neuroscience and Medicine (INM-6), Computational and Systems Neuroscience, Research Center Jülich, Germany; 5 Laboratory for Statistical Neuroscience, RIKEN Brain Science Institute, Wako City, Japan

## 

Ever since the discovery of precisely timed events of cortical neurons [1], their role for information processing has been highly debated. The widespread belief that synchrony is an epiphenomenon caused by shared afferents among neurons [2] has constantly been challenged by reports observing task related modulation of synchrony, lately in primary visual cortex [3] and motor cortex [4]. More so, the recently found decorrelation in cortical networks [5] suggests that the ground state of recurrent balanced networks provides a suitable substrate on top of which synchronized events can represent information.

In this work we theoretically investigate to what extent common synaptic afferents and synchronized inputs each contribute to closely time-locked spiking activity of pairs of neurons [6]. We employ direct simulation and extend earlier analytical methods based on the diffusion approximation [7] to pulse-coupling, allowing us to introduce spiking correlations in the afferent synaptic activity. We compare situations in which the covariance in the input to a pair of model neurons is kept constant, but is realized by different proportions of common afferents and spiking synchrony. This allows us to address the question how much synchrony is caused by afferent synchronized events and how much is intrinsic to cortex due to its structure.

**Figure 1:**
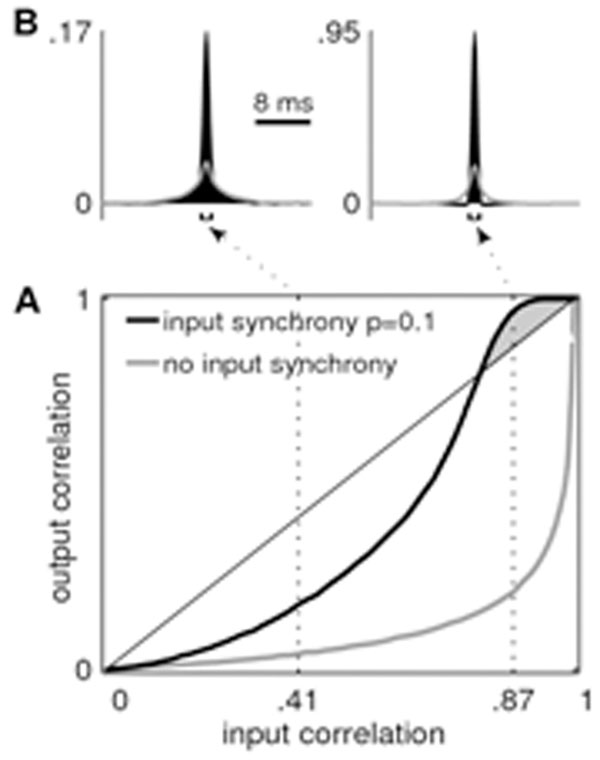
Correlation transmission of a pair of model neurons. **A** Output correlation at 1 ms precision as a function of input correlation. Transmission for 10 per cent pairwise synchronous input spikes in black, no input synchrony in gray. The correlation gain exceeds unity in the gray shaded area. **B** Time-resolved correlation functions. Same gray code as in A.

We find that at fixed input covariance, already weakly synchronous inputs boost the synchrony in the outgoing spiking activity compared to shared input alone (Fig. 1A), sharpening the correlation functions (Fig. 1B). In the regime of strong input synchrony we observe that the output correlation becomes even higher than the correlation in the input (cf. gray area in Fig. 1A). Recent theoretical insights [8] into the non-linear response properties of neurons enable us to explain how such correlation transmission gain > 1 is possible.

Partially supported by BMBF Grant 01IB001A (brain@work), the Helmholtz Alliance on Systems Biology, the Next-Generation Supercomputer Project of MEXT, EU Grant 15879 (FACETS), EU Grant 269921 (BrainScaleS). All network simulations carried out with NEST (http://www.nest-initiative.org).
